# Gastroesophageal reflux in lockdown

**DOI:** 10.2144/fsoa-2023-0042

**Published:** 2023-05-08

**Authors:** Hafez Al-Momani, Dua'a Al Balawi, Muna Almasri, Hadeel AlGhawrie, Lujain Ibrahim, Lena Adli, Hadeel Al Balawi, Sameer Al Haj Mahmoud

**Affiliations:** 1 Department of Microbiology, pathology & forensic medicine, Faculty of Medicine, The Hashemite University, Zarqa, 13133, Jordan; 2 Faculty of Applied Medical Sciences, The Hashemite University, Zarqa, 13133, Jordan; 3 Infection control officer, Infection Control Unit, King Hussein Cancer Center, Amman, 11941, Jordan; 4 Department of Basic Medical Science, Faculty of Medicine, Al-Balqa’ Applied University, AL-Salt, 19117, Jordan

**Keywords:** COVID-19, COVID-19 lockdown, gastroesophageal reflux, gastroesophageal reflux disease, gastrointestinal system

## Abstract

**Aim:**

This study examines the changes in gastroesophageal reflux disease (GERD) symptom frequency among patients with GERD throughout the COVID-19 pandemic.

**Methods:**

A structured questionnaire was distributed among 198 GERD patients. The questionnaire consisted of a demographic characteristic assessment, the GerdQ questionnaire, and a reflux symptom index (RSI) questionnaire.

**Result & conclusion:**

A statistically significant increase in GerdQ score was identified among participants during the COVID-19 pandemic (t = 7.055, df = 209, p < 0.001), who had experienced an increase in the frequency of positive predictors of GERD and a decrease in the frequency of negative predictors of GERD. The COVID-19 pandemic and its related lockdown countermeasures may have led to exacerbating and worsening GERD symptoms.

## Plain language summary

There is a lack of decisive research into the impacts of the COVID-19 pandemic and its lockdown countermeasures on patients with GERD. We investigated the changes in symptomatic frequency among GERD patients in Jordan during the COVID-19 pandemic in a cross-sectional study involving 198 GERD patients. A statistically significant number of participants experienced an increase in the frequency of positive predictors of GERD, and a decrease in the frequency of negative predictors of GERD. In addition, the impacts of GERD itself were also found to have increased during the pandemic, with patients struggling to sleep or attain additional medication to treat their condition.

The virus underlying COVID-19, SARS-CoV-2, has caused over 98 million confirmed cases and 2.2 million deaths since January 2020. SARS-CoV-2 induces a severe acute respiratory syndrome by entering the cells of an infected individual mainly via the ACE2 cell receptors [1–4].

Previous studies have shown that the oral and nasal mucosa, the nasopharynx, the esophagus, the lung, the small intestine, the colon, the kidney, the spleen, the liver, and the brain all contain ACE2 [3,4]. In addition, it has been observed that ACE2 expression is around 100-fold greater in the digestive system than in the respiratory system [5]. Since there are a number of ACE2-expressing organs in the digestive tract, it stands to reason that SARS-CoV-2 [6] may potentially infect these organs and influence gastrointestinal (GI) comorbidity in some way. Although fever, cough, and dyspnea are the most common symptoms associated with COVID-19 [7], patients with a clinically significant subgroup of COVID-19 also report GI problems, which are commonly accompanied by increased liver enzymes [8]. COVID-19 has been observed to manifest first as GI symptoms in some patients [9]. These results imply that the virus may affect the digestive tract, which may account for the wide variety of GI symptoms seen in COVID-19.

“Comorbidity” refers to any long-term health condition that coexists in an individual with a specific condition of interest; the importance of comorbidity in modifying severity and outcomes in COVID-19 was recognized in the earliest scientific reports [10]. Diabetes, hypertension, obesity, and a history of ischemic heart disease were often reported as co-morbidities among the COVID-19 patients [11]. One such group of comorbidity are those who have diseases and symptoms related to the GI tract, which have been found to increase with the COVID-19 pandemic by a number of epidemiological studies [12–14].

Of the various types of GI disorders, gastroesophageal reflux disease (GERD) is considered to be among the most common, caused by a backflow of stomach contents and inducing symptoms and complications in that region that may include regurgitation and heartburn [15–17]. Laryngopharyngeal reflux (LPR) is a variant of GERD, wherein this backflow of stomach contents reaches the laryngopharynx [18]. The distinction between LPR and GERD has become increasingly more defined over the last 25 years, with patients with LPR presenting unique symptoms, reflux patterns, and responses to treatment than their counterparts with GERD [18].

Increased risk for GERD has been associated with lifestyle variables such as being overweight, low level of physical activity, dietary habit, smoking, alcohol intake and sleeping posture [19]. Increased intake of fatty meals and certain beverages, such as tea, coffee, and fizzy drinks, are dietary contributors [20]. Quality of life, job performance, and sleep are all negatively impacted by GERD [21,22]. In addition, research shows that it has serious economic consequences [23].

Though further research is needed, observational links have already been drawn between GERD and COVID-19. Both diseases share similar major risk factors, such as obesity and smoking [24], and develop similar symptoms among patients [25]. A genetic correlation has also been found between patients hospitalized with COVID-19 and their susceptibility to GERD, as shown by Mendelian randomization findings that have suggested a causal link between the two diseases [26]. The prevalence of GERD increased from 24.8% before the pandemic to 34.2% during the pandemic, according to research conducted in Saudi Arabia [27]. There was an increase in the severity of GERD symptoms during the epidemic as well as increase the frequency of administration of GORD medications [27].

Despite the large body of research already existing on the negative impacts of lockdown countermeasures to COVID-19 on the health of patients suffering from chronic diseases, including diabetes, hypertension, and a range of mental health issues and disorders as well [28,29]. To our knowledge, only a few of the previous studies [26,27] exist that seek to address the same impacts on patients suffering from GERD. This study therefore aims to rectify this absence of literature by taking the following steps: 1) assessing the impacts of the COVID-19 pandemic and its lockdown countermeasures on patients suffering from GERD; and 2) determining the prevalence of LPR among patients diagnosed with GERD, taking the country of Jordan as a geographic basis for this study. If a link were to exist between GERD and COVID-19, we might expect to find a correlated increase or decrease in GERD symptoms during the COVID-19 pandemic. It is therefore hoped that this study might provide a complementary perspective on what links these diseases, if indeed such a link exists and may be identified.

## Methods

### Study design

A cross-sectional study was undertaken with structured questionnaires distributed among known cases of GERD patients who attend the upper GI clinic at Prince Hamza Hospital. From January through May of 2021, data was gathered during the second wave of the COVID-19 pandemic in the country of Jordan. A second, more intense wave of COVID-19 transmission, prompted by the quickly dispersing UK strain, started around the middle of January, and peaked around the middle of March of the same year [30].

All patients with confirmed GERD who attend the upper GI clinic at Prince Hamza Hospital for follow-up and medication were approached. Each patient's medical history is reviewed before to enrollment to confirm the diagnosis of GERD, which is often achieved by a combination of clinical symptoms, responsiveness to acid suppression, and objective testing using upper endoscopy and esophageal pH monitoring.

In this study, we included adults aged 18 years or older who were diagnosed with GORD. Patients with previous GI surgery or malignancy, mental illness, who did not give their consent and were less than 18 years old, were excluded. Pregnant females were also excluded. Patients who had been previously infected with SARS-coV- virus in the past 2 months were excluded from this study as this study explored the impact of the pandemic and its lockdown countermeasures on patients suffering from GERD. All eligible patients who signed a permission form were interviewed, their hospital stays were monitored, and their data was included in the study.

Potential participants met with a research assistant and were given information related to the research. They were assured that the data they provided would remain strictly confidential and would only be shared among study conductors and that no personal information would be required of them. Potentially all patients from the population of the regional clinic who visited the clinic while the research was being conducted were contacted (there were a total of 238), and we were able to enroll 83.5% of all the eligible patients. 198 patients answered the questionnaire, all of whom were adults who had been diagnosed with GERD in the past and who took regular dosages of proton pump inhibitors to ameliorate the symptoms of their condition.

### Data collection methods

An online self-administered questionnaire used by this study for data collection was distributed via WhatsApp among participants and validated in a pilot study of 21 participants (the responses of which have been excluded from subsequent analysis). The questionnaire consisted of three parts:

The first section of the questionnaire acted as a preliminary means of assessing demographic characteristic of the participants and asked questions pertaining to each participant's age, gender, marital status, tobacco and alcohol use, as well as their height (in meters) and weight (in kilograms) with which to determine their BMI (kg/m).

The second section of the questionnaire pertaining to GerdQ scores (Table 1) requested that participants score the occurrence of their symptoms over a week as well as detail any medications they had taken within this period. A four-point graded Likert scale (0–3) was used to score the frequency of four specific positive predictors, those being heartburn, regurgitation, sleep disturbance owing to reflux symptoms, and the frequency of over the counter (OTC) medication usage to counteract reflux symptoms. A 4-point graded reversed Likert scale (3–0) was also used to score the frequency of two specific negative predictors, epigastric pain and nausea. Numerical responses to these answers allowed for a total GerdQ score ranging from 0 to 18. Qualifiers pertaining to sleep disturbance and OTC medication usage were also used to assess the impact of GERD on each participant, providing a separate ‘impact score’ ranging from 0 to 6. The GerdQ questionnaire was found to have a sensitivity of 65% and a specificity of 71% for the accurate diagnosis of GERD [31]. Participants must provide two answers to each question within its second section pertaining to GerdQ scores; the first to reflect their status prior to the COVID-19 pandemic, and the second to reflect their status in the present and moment of their response.

**Table 1.** Gastroesophageal Reflux Disease Questionnaire (GerdQ).

Question	Frequency score (points) for symptom			
	0 day	1 day	2–3 day	4–7 day
How often did you have a burning feeling behind your breastbone (heartburn)?	0	1	2	3
How often did you have stomach contents (liquid or food) moving upwards to your throat or mouth (regurgitation)?	0	1	2	3
How often did you have pain in the center of the upper stomach?	3	2	1	0
How often did you have nausea?	3	2	1	0
How often did you have difficulty getting a good night's sleep because of your heartburn and/or regurgitation?	0	1	2	3
How often did you take additional medication for your heartburn and/or regurgitation, other than what the physician told you to take) (such as Tums, Rolaids, Maalox?)	0	1	2	3

Respondents provided frequency scores after reflecting on their symptoms from the previous week.

The third section of this questionnaire included the reflux symptom index (Table 2) that was used in order to assess the presence and intensity of commonly reported LPR symptoms [32]. This index allows participants to grade their experiences on a 0–5 scale, with 0 describing minimal or non-existent symptoms and 5 describing severe and pronounced symptoms. This index was used to gather a range of symptoms, with participants asked to respond to the following list of complaints: hoarseness or a problem with your voice; clearing your throat; excess throat mucus or postnasal drip; difficulty swallowing food, liquids, or pills; coughing after you ate or after lying down; breathing difficulties or choking episodes; a troublesome or annoying cough; sensations of something sticking in your throat or a lump in your throat; and heartburn, chest pain, indigestion, or stomach acid coming up. Scores greater than 13 were considered clinically and statistically significant as indicators of LPR [33]. This index scored highly in both test-retest reliability (rs = 0.921) and internal consistency reliability (α = 0.969) [34].

**Table 2.** The reflux symptom index .

Within the last month, how did the following problems affect you? 0 = no problem; 5 = severe problem						
	0	1	2	3	4	5
1. Hoarseness or a problem with your voice						
2. Clearing your throat						
3. Excess throat mucus or postnasal drip						
4. Difficulty swallowing food, liquid, or pills						
5. Coughing after you ate or after lying down						
6. Breathing difficulties or choking episodes						
7. Troublesome or annoying cough						
8. Sensation of something sticking in your throat or a lump in your throat						
9. Heartburn, chest pain, indigestion, or stomach acid coming up						
Total RSI score >13 = Abnormal)						

Respondents enter severity scores after reflecting on their symptoms from the previous month.

RSI: Reflux symptom index.

### Statistical analysis

GraphPad InStat 6.0 was used to analyze the data collected in this study, with descriptive measures used for categorical data, including counts and proportions presented as mean, standard deviation (SD), and percentages. The bootstrapping method calculated 95% of Cls in Cramer's V statistics. A two-sided p-value of ≤0.05 was considered statistically significant, with chi-square used to assess the significance of variables pertaining to GERD. Paired t-tests were also employed to find the differences between any two variables on the same subject.

## Results & findings

### Patients characteristic

As is shown in Table 3, of the 198 participants who responded to this questionnaire, 58.1% were male and 41.9% were female. Participants fell most commonly into the age bracket of 38–48 years old at 34.3%, followed by 29.8% in the bracket of 28–38, 14.1% in the bracket of 18–28, 13.6% in the bracket of 48–58, and 8.1% in the bracket of 58 and above. Regarding marital status, 60.1% reported that they were married, with the remaining 39.9% reporting that they were single or divorced. Most patients were found to be overweight, as determined by a mean BMI of 27.0 ± 4.5, while 28 (14.1%) were found to have at least one identifiable comorbid condition, the most common of which being hypertension (8.1%), diabetic mellitus (7.1%), and cardiovascular disease (6.6%). Finally, thirty-six participants (18.2%) identified themselves as nonsmokers.

**Table 3.** Basic demographic and medical information of the patients included in this study.

Characteristic	All GERD patient cohort (n = 198)
Age group (years)	
18–28	28 (14.1%)
28–38	59 (29.8%)
38–48	68 (34.3%)
48–58	27 (13.6%)
58–68	16 (8.1%)
>68	0
BMI mean ± SD (range)	27.0 ± 4.5 (18–35)
Gender	
Male	115 (58.1%)
Female	83 (41.9%)
Marital status	
Married	119 (60.1%)
Single or divorced	79 (39.9%)
Underlying medical conditions	
At least one comorbid condition	28 (14.1%)
Chronic cardiovascular disease	13 (6.6%)
Chronic lung disease	10 (5.1%)
Diabetes mellitus	14 (7.1%)
Hypertension	16 (8.1%)
Gastrointestinal disease	5 (2.5%)
Renal disease	3 (1.5%)
Other comorbidity	2 (1.0%)
Current tobacco use	36 (18.2%)
Current alcohol use	11 (5.6%)

GERD: Gastroesophageal reflux disease; SD: Standard deviation.

**Figure 1.**
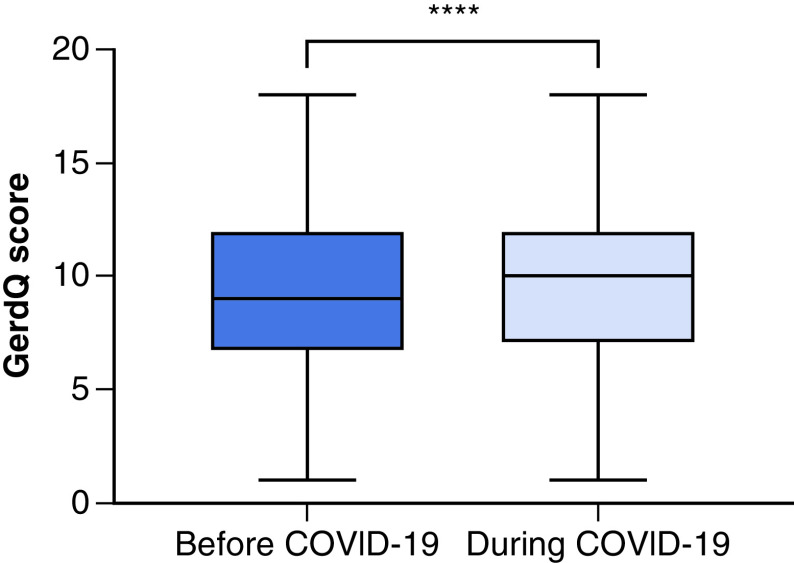
The difference between GerdQ scores reported by patients prior to COVID-19 and those reported during COVID-19. ****p-value < 0.0001.

**Figure 2.**
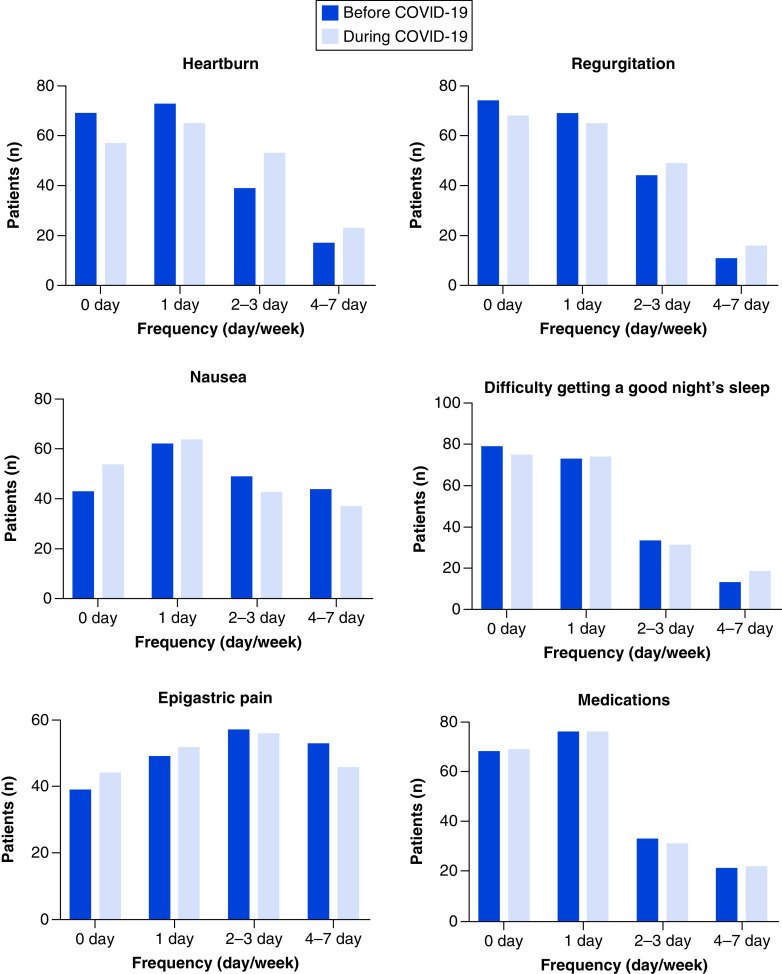
A comparison of patient responses to different items of GerdQ scoring prior to the COVID-19 pandemic and the changes that occurred during the COVID-19 pandemic. The x-axis represents the frequency of items in terms of days per week. The y-axis represents the number of patients reporting each item.

A statistically significant increase in GerdQ score was identified among participants during the period of the pandemic, as is demonstrated in Figure 1 (t = 7.055, df = 209, p < 0.001). Figure 2 demonstrates the increase in positive predictors of GERD that was identified in this study, including predictors such as heartburn and regurgitation, and the reduced frequency of counteractively negative GERD predictors. The aforementioned ‘impact score’ also demonstrated an increased impact of GERD symptoms during this period, such as through an increased reliance on medication by GERD patients and a difficulty for such patients to maintain a consistent and restful sleep cycle. As may be seen in Table 4, 8.6% of participants reported incidences of heartburn and 5.6% of regurgitation at a frequency of 4–seven-times per week prior to the pandemic. These results increased to 11.6 and 8.1%, respectively, when participants were asked to report on the frequency of these symptoms during the pandemic itself. In terms of negative predictors of GERD, 26.8% and 22.2% of participants reported incidences of epigastric pain and nausea at a frequency of 4–seven-times per week prior to the pandemic. This result decreased to 23.2% and 18.7% when participants were asked to report on the frequency of this negative predictor during the pandemic itself. Lastly, GERD patients also reported an increasingly poor quality of sleep during the pandemic, as well as an increase in the use of additional medication to combat their symptoms.

**Table 4.** Patient responses to GerdQ for each item in terms of the number and percentage of patients prior to COVID-19 and the changes that occurred to symptomatic frequency during the COVID-19 pandemic.

Question	Frequency score (points) for symptom before COVID-19				Frequency score (points) for symptom during COVID-19			
	0 day, n (%)	1 day, n (%)	2–3 day, n (%)	4–7 day, n (%)	0 day, n (%)	1 day, n (%)	2–3 day, n (%)	4–7 day, n (%)
How often did you have a burning feeling behind your breastbone (heartburn)?	69 (34.8%)	73 (36.9%)	39 (19.7%)	17 (8.6%)	57 (28.8%)	65 (32.8%)	53 (26.8%)	23 (11.6%)
How often did you have stomach contents (liquid or food) moving upwards to your throat or mouth (regurgitation)?	74 (37.4%)	69 (34.8%)	44 (22.2%)	11 (5.6%)	68 (34.3%)	65 (32.8%)	49 (24.7%)	16 (8.1%)
How often did you have pain in the center of the upper stomach?	39 (19.7%)	49 (24.7%)	57 (28.8%)	53 (26.8%)	44 (22.2%)	52 (26.3%)	56 (28.3%)	46 (23.2%)
How often did you have nausea?	43 (21.7)	62 (31.3%)	49 (24.7%)	44 (22.2%)	54 (27.3%)	64 (32.3%)	43 (21.7%)	37 (18.7%)
How often did you have difficulty getting a good night's sleep because of your heartburn and/or regurgitation?	79 (17.6)	73 (27.6%)	33 (33.8%)	13 (21.0%)	75 (11.4%)	74 (37.4%)	31 (15.7%)	18 (9.1%)
How often did you take additional medication or increase the dose for your heartburn and/or regurgitation, other than what the physician told you to take)	68 (34.3%)	76 (38.4%)	33 (16.7%)	21 (10.6%)	69 (34.8%)	76 (38.4%)	31 (15.7%)	22 (11.1%)

### Prevalence of LPR

**Figure 3.**
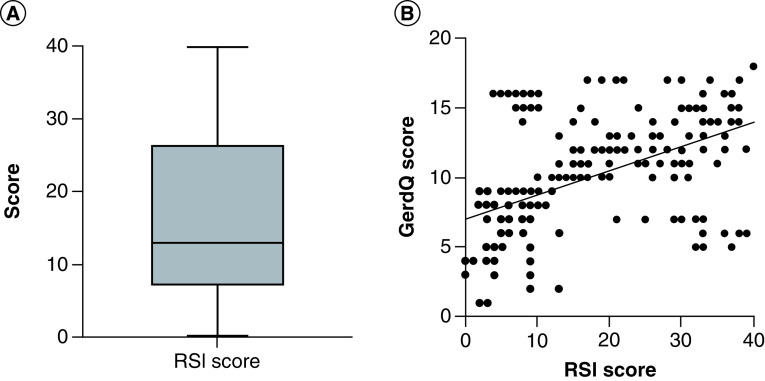
Reflux symptom index score and its correlation with GerdQ score among the patients cohort. **(A)** RSI score among the participants, median = 7.0 with mean ± SD = 16.3 ± 11.4. **(B)** Correlation between the RSI and GerdQ scores among participants (R = 0.49, 95% CI (0.38 to 0.59), p < 0.0001). RSI: Reflux symptom index; SD: Standard deviation.

Of the 198 participants in this study, ninety-five provided responses amounting to an RSI score of more than thirteen, indicating the possible presence of LPR. RSI scores range from 0–40, with a mean score being found across all respondents of 17.1 ± 11.9 and a median score of 14.5 (Figure 3A). A statistically significant positive correlation was identified between the RSI and GerdQ scores provided by participants (R = 0.49, 95% CI (0.38 to 0.59), p < 0.0001) (Figure 3 B), with a mean GerdQ score of 7.8 ± 4 found among all participants with an RSI score below 13 compared with the mean GerdQ score of 11.7 ± 3.4 found among all participants with an RSI score of 13 or higher. No other social demographics or comorbid symptoms were found to be statistically significantly different between participants with an RSI score below 13 and participants with an RSI score of 13 or above (Table 5).

**Table 5.** The difference between GERD patients who have RSI scores below 13 and those who have RSI scores greater than thirteen in terms of the GerdQ score, demographics and previous medical history.

	RSI below 13 (103, 52.0%)	RSI score more than 13 (n = 95, 47.9%)	p-value
Mean ± SD of GerdQ score (during COVID pandemic)	7.8 ± 4	11.7 ± 3.4	<0.0001
Age group (years)			
18–28	17 (16.5%)	11 (11.6%)	0.5505
28–38	29 (28.1%)	30 (31.6%)	0.7624
38–48	33 (32.0%)	35 (36.9%)	0.3088
48–58	15 (14.6%)	12 (12.6%)	0.5611
58–68	9 (8.7%)	7 (7.4%)	0.8126
>68	0 (0.0)	0 (0.0)	
BMI mean ± SD (range)	26 + 4.7	27.8 + 4.3	0.8126
Sex			
Male	56 (54.4%)	59 (62.1%)	0.2642
Female	47 (45.6%)	36 (37.9%)	
Marital status			
Married	61 (59.2%)	58 (61.1%)	0.8871
Single or divorced	42 (40.8%)	37 (38.9%)	
Underlying medical conditions			
Chronic cardiovascular disease	7 (6.8%)	6 (6.3%)	0.47
Chronic lung disease	6 (5.8%)	4 (4.2%)	0.78
Diabetes mellitus	8 (7.8%)	6 (6.3%)	0.7602
Hypertension	9 (8.7%)	7 (7.4%)	0.25
Gastrointestinal disease	3 (2.9%)	2 (2.1%)	0.68
Renal disease	2 (1.9%)	1 (1.1%)	0.3625
Other comorbidity	1 (1.0%)	1 (1.1%)	0.77
Current tobacco use	17 (16.5%)	19 (20.0%)	0.19
Current alcohol use	5 (4.9%)	6 (6.3%)	0.47

RSI: Reflux symptom index; SD: Standard deviation.

## Discussion

The purpose of this study is to determine the impact of the COVID-19 pandemic or the restrictive lockdown countermeasures during the pandemic on patients with GERD. This study has also sought to discuss the prevalence of LPR among the cohort of patients diagnosed with GERD in one of Jordanian hospitals. To arrive at a competent analysis of this topic, this study has gathered data from 198 residents of Jordan who have previously been diagnosed with GERD. Most of these participants had complaints about certain characteristic symptoms of GERD, including heartburn and regurgitation, which they claimed had increased in frequency throughout the length of the pandemic. Respondents also claimed that their sleep patterns had suffered because of COVID-19 and that they now relied more on other medications and increased the dose of their medications than they had prior to the lockdown. The findings of this study agree with those of Oliviero, Ruggiero, D'Antonio *et al.* [35] and Lee, Huo, Huang [36], which concluded that COVID-19 had exacerbated certain symptoms of GI in affected individuals, such as functional dyspepsia and irritable bowel syndrome.

This study has found that GERD symptoms became more frequent with the advent of the COVID-19 pandemic, which is consistent with a previous study published by Alhuzaim, Alotaibi [27]. This increase in symptomatic frequency might possibly be attributed to the restricted living styles enforced by COVID-19 lockdown countermeasures and the induced behavioral shifts as a result, such as an increase in coffee ingestion and smoking, which have both been found to have occurred as a result of these measures [37]. Further behavioral changes might also include increased eating and lying down or dietary changes such as consuming a larger lunch as opposed to the smaller lunches provided by offices or commercial cafes in the city [38]. These dietary and behavioral changes may have had adverse effects on the reflux symptoms of GERD patients as a result [39]. A Systematic Review of Longitudinal Studies by González-Monroy *et al.* [40] described a certain change in the public's eating behaviors during the COVID-19 pandemic toward snacking and a preference for sweets and ultra-processed food stuffs as opposed to fruits, vegetables and fresh food, with a decline in the general preference for healthy dietary behaviors in the process. The international consumption of alcohol has also been found to have increased over the course of the pandemic [40], leading to this study's opinion that the general risk of reflux has increased as a predisposing factor for GERD.

It is also apparent that weight plays a fundamental mechanical role in increasing reflux symptoms among patients diagnosed with GERD [41]. Previous research has found a statistically significant increase in the weight of some people during the pandemic [42], with a paper by Bakaloudi *et al.*[43] demonstrating that lockdown measures have negatively impacted the body weights of individuals across the globe. Further studies by Chang *et al.* [44] and Pellegrini *et al.* [45] have found a similar inclination toward higher BMI and international body weights throughout the length of the COVID-19 pandemic.

It may be posited that the influential factors behind the increase in weight and BMI around the world have also played a part in the epidemic of GERD itself. Evidence has shown that more fat-rich and substantial diets may have a certain effect on the prevalence of GERD diagnoses within a general population [46]. Furthermore, high calorific diets have been shown to also increase one's exposure to esophageal acid [46,47]. As such, we may assume that it is likely that the same dietary habits might promote the development of both diseases. In addition, one likely explanation is that the increased weight of an individual places more substantial pressure on the abdomen [48] which, in turn, may increase the risk of unwanted muscular relaxation in the lower esophageal sphincter (this is the ring of muscle between the esophagus and the stomach, known as the ‘LES’).

Furthermore, many studies demonstrated the psychological effects of mandatory quarantines during past pandemics have on people. Quarantined patients in Toronto during the 2002–2004 SARS epidemic who responded to a web-based survey by Hawryluck, Gold, Robinson, Pogorski, Galea, Styra [49] indicated a significant incidence of psychological distress. During the time of isolation, 7.6% of the 1656 patients who had encountered MERS patients reported experiencing anxiety, as reported by Jeong, Yim, Song *et al.* [50]. Recent research based on an online survey by Mazza, Ricci, Biondi *et al.* [51] on 2766 Italians during the COVID-19 epidemic found that the acquaintance of COVID-19 infection was linked to high levels of stress. Sensitivity to reflux might be brought on by stress [52]. Anxiety, especially health-related anxiety, has been shown to lower a person's pain threshold, making them more sensitive to reflux episodes [53]. Another way that stress contributes to reflux is by making individuals swallow more air than usual during the day [54].

This study has found that ninety-five of its 198 participants (47.9%) demonstrated an RSI score exceeding thirteen, indicating the presence of LPR. If this statistic may be extrapolated to describe the GERD population of Jordan, whereby some 1-in-2 individuals affected by GERD may also be affected by LPR, this would suggest that LPR is common and prevalent in the region and that further studies are needed for more accurate statistics. Moreover, this would also have large-ranging ramifications for both the primary and secondary care resources of the country, such as in general physician and clinic visits, treatment costs, and investigation frequency. LPR-related symptoms, which may include hoarseness and foreign body sensations in the throat, may also create a heightened sense of anxiety among patients, who may fear they have an undetected malignancy. More often than not, said individuals will be eventually referred to an otolaryngologist for diagnosis and reassurance, which - if such referrals begin to exceed nominal levels - may begin to put unwanted pressure upon Jordanian's already strained medical budget and healthcare system, when in fact these patients may simply be treated with PPI and liquid alginates instead.

## Limitation

While conducting this study, we remain aware that using an RSI >13 as a diagnostic evaluation of LPR is potentially problematic. RSI may induce a number of nonspecific symptoms, such as hoarseness, coughing, throat-clearing, and a build-up of sticky mucus, that may also be related to many other common inflammatory diseases of the upper aerodigestive tract, such as allergies, rhinitis, chronic rhinosinusitis, and pharyngolaryngitis [55–57]. Diagnosing for LPR in the absence of the gold standard of either pH manometry or the combination of laryngoscopy and detected LRP symptoms therefore relies on such simplified RSI scoring systems, which is considered one limitation of this study. This limitation, while it introduced a speculative element of subjectivism to the hypothetical diagnosis of the study's respondents, is nevertheless considered the best possible approach that is usually used to separate out diagnoses of LPR from general GERD [58].

Indeed, subjectivism is one of the more fundamental issues pervading this study, as it relies solely upon data gathered from the opinions and memories of its questioned individuals. Without a secondary analytical perspective gathered from a more specific investigative method, such as flexible endoscopy or ambulatory 24-hour double-probe pH monitoring, the accuracy of this study's results may also be called into question. However, as more specific investigative approaches are highly costly and invasive, they may also be considered unsuitable foruse in such a study. A third limitation of this study may be found in the size of its sample group, which may be considered relatively small. This might limit the study's generalizable capabilities to be extracted to a more international conclusion on the epidemic's impacts on GERD, such that larger studies investigating multiple treatment centers are advised to improve the validity of this study's findings on a larger scale. Finally, as this study retrieved data through a survey questionnaire, individual response and recall bias may also have played a role in the accuracy of its findings, which cannot be managed or controlled by the study's researchers. Despite these caveats, this research is one of the few to examine how the COVID-19 pandemic has affected the prevalence of GORD-19 symptoms.

## Conclusion

To conclude, this study found an increase in symptomatic frequency among GERD patients, which seems to correlate with the development of the COVID-19 pandemic and its related social and legislative countermeasures, as well as the behavioral shifts that have come because of these measures as well. To further understand the pandemic's impact on GERD, we need a bigger multiregional sample. The findings of this work need to be evaluated and confirmed by future studies. This research might help gastroenterologists and other doctors to identify areas that could help in reducing the severity of GORD among population in future pandemic or other lockdown measures. Since this study identified a significant frequency of LPR among individuals diagnosed with GERD Further research is recommended to validate these results and to appropriately extrapolate them onto a broader national scale.

**Summary points**

- Previous studies have investigated the links between COVID-19 and the exacerbation of certain comorbid conditions. However, there remains a lack of decisive research into the impacts of the COVID-19 pandemic and its lockdown countermeasures on patients suffering from gastroesophageal reflux disease (GERD).
- This study therefore looks to examine the changes in symptomatic frequency among patients of GERD throughout the COVID-19 pandemic in the country of Jordan, as well as to evaluate the prevalence of laryngopharyngeal reflux (LPR) among GERD patients in the region.
- A statistically significant number of participants had experienced an increase in the frequency of positive predictors of GERD and a decrease in the frequency of negative predictors of GERD.
- The impacts of GERD itself were also found to have increased during the pandemic, with patients struggling to sleep or attain additional medication for GORD.
- This study concludes that the COVID-19 pandemic and its related lockdown countermeasures may have led to a change in GERD patient lifestyle, exacerbation and worsening of their symptoms as a result. Further research is needed on this subject to arrive at a more definitive result.
